# Sugar-induced cell death (SICD) in *Saccharomyces cerevisiae*: insights into nitrogen-mediated rescue and apoptotic cell death pathways

**DOI:** 10.15698/mic2026.05.874

**Published:** 2026-05-11

**Authors:** Raveena Parbhudayal, Hai-Ping Cheng

**Affiliations:** Department of Biological Sciences, Lehman College, The City University of New York, Bronx, NY 10468, USA; The Graduate Center, City University of New York, New York, NY 10016, USA

**Keywords:** sugar-induced cell death, apoptosis, cell death, yeast, nitrogen rescue, glucose metabolism

## Abstract

Sugar-induced cell death (SICD) is a phenomenon observed in *Saccharomyces cerevisiae* whereby cells rapidly lose viability in glucose-only solutions. One theory suggests that SICD occurs due to an imbalance in nitrogen and carbon, however, limited studies are available to support this. When stationary phase cells are transferred to glucose-only solutions, cell death resembles that of apoptosis, while exponential phase cells show hallmarks of primary necrosis. Apoptosis in stationary phase cells is independent of the yeast metacaspase, *YCA1*, however, it remains unknown if SICD occurs through a caspase-independent pathway. Using stationary phase *S. cerevisiae* BY4741, we showed that SICD can be induced to the same degree with 10 mM or 110 mM glucose. Interestingly, SICD induced by 10 mM of glucose can be protected by supplementation with low concentrations of highly preferred organic nitrogen sources, namely glutamate, glutamine, and arginine, as well as high concentrations of non-preferred organic nitrogen sources. Additionally, cell death can be rescued by deletion of *YCA1* and genes involved in caspase-independent apoptosis—*STE20, NMA111, AIF1,* or *NUC1*. On the other hand, when *S. cerevisiae* BY4741 is challenged with 110 mM glucose, SICD can only be rescued by supplementation with the same preferred organic nitrogen sources or deletion of *AIF1* or *NMA111.* In all cases, protection is associated with a decrease in intracellular ROS and preservation of membrane integrity. Taken together, 110 mM glucose results in a catastrophic cell death phenotype that is more difficult to rescue, and nuclear localization of Aif1p and Nma111p is important for cell death in response to glucose.

## INTRODUCTION

Sugar-induced cell death (SICD) in yeast is a phenomenon whereby cells rapidly lose viability in the presence of glucose and the absence of any other nutrients 1–3. SICD is dependent on the entry of glucose into the cell and phosphorylation to glucose-6-phosphate 1, 2, 4. Both metabolized and non-metabolized carbon sources can induce SICD 2, 5. SICD analyses are done mainly with 2% glucose concentrations, although it can be induced by even 0.1% glucose 2, 4. Additionally, SICD is mainly studied in *Saccharomyces cerevisiae,* however, it also occurs in *Candida spp.* and was found to be highly dependent on temperature 5, 6. Glucose incubation also results in dramatic acidification of the extracellular environment, which has been suggested to occur because of activation of H ${}^{+}$ -ATPases and an increase in membrane potential, which can be countered by addition of KCl 5, 7–9. Supplementation with phosphate was also shown to protect against SICD by replenishing intracellular phosphate reserves and preventing ATP exhaustion in the absence of additional nutrients 10.

When stationary phase cells of *S. cerevisiae* are transferred to glucose-only solutions, cell death is accompanied by DNA and RNA degradation, membrane blebbing, membrane fragmentation, vesicle formation, and nuclear fragmentation - all hallmarks of apoptosis 3. However, cell death is independent of the only found yeast metacaspase, *YCA1* 7. Yeast can undergo caspase-dependent and caspase-independent cell death, however, the implication of caspase-independent cell death pathways in SICD remains to be explored. Δ *ste20* was found to be resistant to SICD 11. *STE20* encodes a kinase, Ste20p, which translocates to the nucleus in a caspase-independent manner and phosphorylates histone H2B during H ${}_{2}$ O ${}_{2}$ -induced cell death, suggesting a similar regulatory mechanism among the various types of cell death 11, 12.

On the other hand, SICD of exponential-phase cells results in a death phenotype that resembles primary necrosis. Cell death is induced within 15 minutes, with evidence of membrane damage, swollen nuclei, chromatin release in the cytoplasm, absence of phosphatidylserine externalization, and is independent of *de novo* protein synthesis and *YCA1* 8, 13, 14.

Additionally, supplementation of non-carbon nutrients protects from SICD, supporting the idea that SICD may be a result of an imbalance in the nitrogen and carbon available for the cells to support growth 15. Like carbon, excess nitrogen was also shown to induce cell death in yeast and shorten the chronological lifespan 16–18. Ammonium sulfate was shown to induce apoptosis followed by secondary necrosis 18. As ammonium supplementation increases, an increase in glucose consumption is observed. Therefore, a balance of nitrogen and carbon is required for cell survival and proliferation 16, 17. To expand on these findings, we tested a range of different nitrogen sources to determine the minimum requirement to prevent cell death in the presence of glucose. We also investigated the role of caspase-dependent and caspase-independent apoptosis cell death pathways in SICD of stationary phase cells.

## RESULTS

### SICD can be prevented by supplementation with complex nitrogen sources

**Figure 1:**
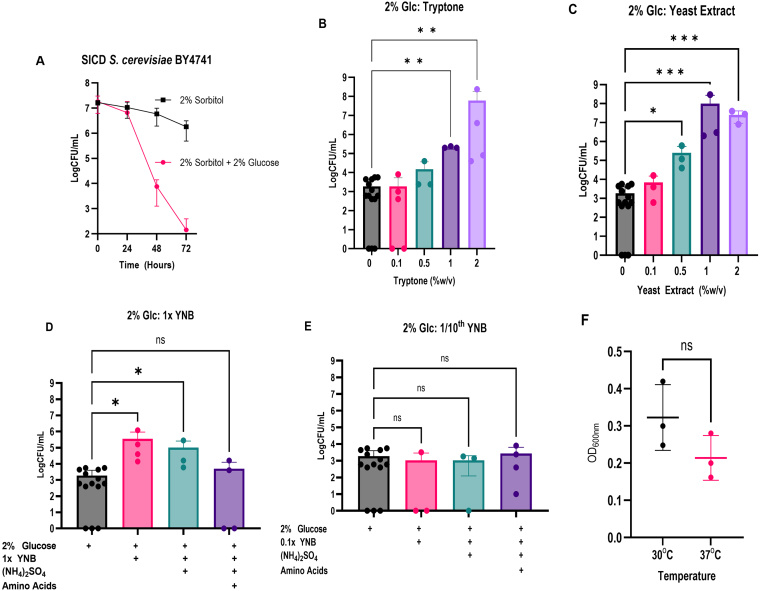
Complex nitrogen sources can protect against SICD. SICD was confirmed in *S. cerevisiae* BY4741 **(A)**. *S. cerevisiae* BY4741 was cultured to stationary phase and then transferred to either 2% glucose only or 2% glucose supplemented with 0.1%, 0.5%, 1% 2% (w/v) tryptone **(B)** or yeast extract **(C)** and 1x **(D)** or 1/10 ${}^{\mathrm{th}}$ YNB medium only **(E)** or YNB supplemented with (NH ${}_{4}$ ) ${}_{2}$ SO ${}_{4}$ with or without amino acids. Survival was determined before incubation and after 72 hours of incubation at 37 ${}^{o}$ C. Growth of *S. cerevisiae* BY4741 was confirmed in YNB medium (with auxotrophic nutrients) at 30 ${}^{o}$ C and 37 ${}^{o}$ C **(F)**. The data shown are mean ± SD. ${}^{*}$ = *p* ≤ 0.04, ${}^{**}$ = *p* ≤ 0.005, ${}^{***}$ = *p* ≤ 0.0009, ns = not significant.

It was previously shown that SICD can be prevented by supplementation of nitrogen in the form of ammonium citrate and amino acids, however, the names of the amino acids were not provided 1, 15. To expand on these findings, we tested a range of different nitrogen sources commonly used to culture yeast in the laboratory to determine the role of nitrogen and carbon balance in SICD. We first confirmed that SICD occurs in *S. cerevisiae* BY4741, the strain used in this study. Consistent with prior studies, the cells survived in sorbitol only, while in the presence of glucose, there was almost a 5-log reduction in viability within 72 hours (Figure 1**A**) 1–5, 7, 8, 11, 14, 15, 19, 20.

To determine the effect of nitrogen sources on SICD, *S. cerevisiae* BY4741 was inoculated in 2% glucose supplemented with 0.1%, 0.5%, 1%, and 2% (w/v) tryptone or yeast extract, which are components of YPD medium used to culture yeast in the laboratory. Viability was determined after 72 hours of incubation. As shown in Figures 1**B** and **C**, 1% and 2% tryptone and 0.5%, 1%, and 2% yeast extract resulted in significant protection against SICD when compared to 2% glucose only (Figures 1**B** and **C**). Similar results were also observed in *S. cerevisiae* S288c-PDA (data not shown). Therefore, our findings support previous studies that SICD can be prevented by supplementation with nitrogen sources 15.

### Increasing nitrogen content in minimal media shows a negative impact on cell survival

To determine the specific nitrogenous compounds that protect against SICD, we asked whether the components of YNB medium are sufficient to protect against SICD. We used three formulations of YNB medium: YNB only, which consists of trace minerals, vitamins, and salts; YNB + (NH ${}_{4}$ ) ${}_{2}$ SO ${}_{\mathrm{4,}}$ and YNB + (NH ${}_{4}$ ) ${}_{2}$ SO ${}_{4}$ + amino acids. The amino acids present were methionine, histidine, and tryptophan, added by the manufacturer. When *S. cerevisiae* BY4741 was inoculated in 2% glucose with 1x YNB only and YNB + (NH ${}_{4}$ ) ${}_{2}$ SO ${}_{4}$ , there was a 2-log difference in viability compared to cells in 2% glucose-only after 72 hours of incubation. However, when YNB + (NH ${}_{4}$ ) ${}_{2}$ SO ${}_{4}$ + amino acids were supplemented to 2% glucose, there was no significant difference in cell viability (Figure 1**D**). We then diluted the YNB formulation to 1/10 ${}^{\mathrm{th}}$ final concentration (Figure 1**E**) and found that neither of the three formulations protected against SICD (Figure 1**E**), suggesting that this concentration of nutrients is too low to sustain survival in 2% glucose.

Taken together, these results suggest that in the presence of 2% glucose, YNB only and YNB + (NH ${}_{4}$ ) ${}_{2}$ SO ${}_{4}$ can protect against SICD. However, the addition of amino acids to YNB + (NH ${}_{4}$ ) ${}_{2}$ SO ${}_{4}$ does not lead to any protection against SICD. *S. cerevisiae* BY4741 is auxotrophic for leucine, methionine, and uracil. It should be noted that experiments with YNB were done with and without auxotrophic nutrients, and the results remained the same (data not shown). Therefore, auxotrophy may not be a contributing factor to cell death in response to glucose.

Since SICD experiments were carried out at 37 ${}^{\circ }$ C, we examined the growth of BY4741 in YNB medium with 2% glucose at 37 ${}^{\circ }$ C. As shown in Figure 1**F**, there was no significant difference in the growth of cells cultured at 30 ${}^{\circ }$ C and 37 ${}^{\circ }$ C, however, final cell density after 48 hours at 37 ${}^{\circ }$ C was lower than at 30 ${}^{\circ }$ C. This suggests that BY4741 can grow at 37 ${}^{\circ }$ C, but may be stressed. It should be noted that in this growth assay, the starting inoculum was OD ${}_{\mathrm{600 nm}}$ 0.01, while during SICD induction, the inoculum was OD ${}_{\mathrm{600 nm}}$ 1.3.

### SICD is concentration-dependent up to 10 mM glucose

**Figure 2:**
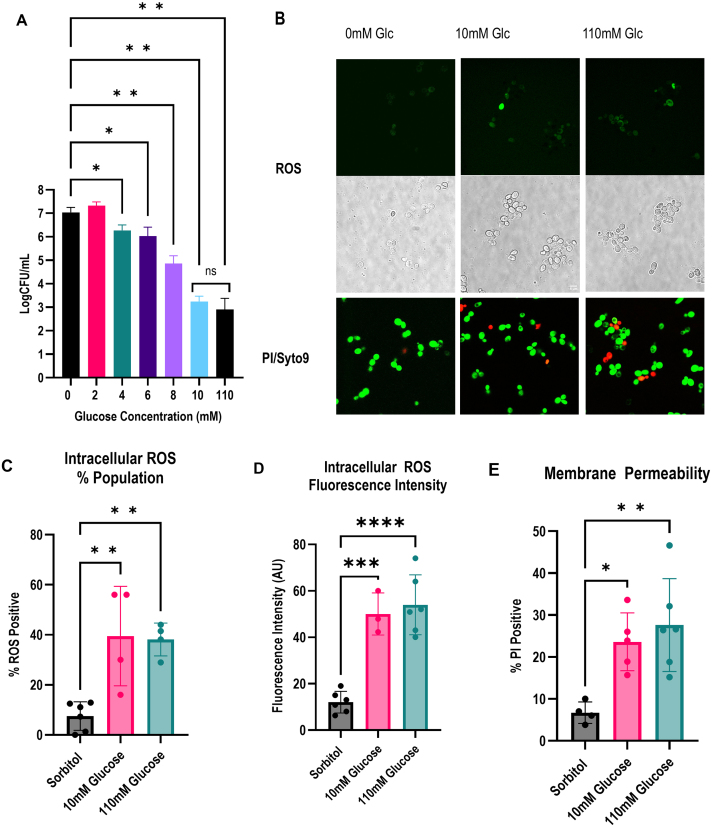
Finding the minimum concentration of glucose required for SICD. Stationary phase cells of *S. cerevisiae* BY4741 were transferred to 2% sorbitol supplemented with 2 mM, 4 mM, 6 mM, 8 mM, 10 mM, and 110 mM glucose, and CFU was determined at 72-hours **(A)**. After 1 hour of incubation, cells in sorbitol (0 mM), 10 mM and 110 mM glucose were stained with H ${}_{2}$ DCFDA to detect accumulation of intracellular ROS (**B**, top and middle brightfield) and PI and Syto9 to detect membrane permeability (**B**, bottom). The images were obtained at 65x magnification and 3x zoom. The percentage of cell staining positive for ROS **(C)** was calculated, and the fluorescence intensity was determined **(D)**. The percentage of cells staining positive for PI were also calculated (E). ${}^{*}$ : *p* ≤ 0.03, ${}^{**}$ : *p* ≤ 0.007, ${}^{***}$ : *p* ≤ 0.0001, ${}^{***}$ : *p* < 0.00001, ns: not significant.

It was previously suggested that low concentrations (0.1% or 5.5 mM) of glucose can cause SICD 2. Therefore, we hypothesized that the protective effect of nitrogen on SICD may be more prominent at lower concentrations of glucose. The minimum glucose concentration required for SICD was determined by testing the effect of 2 mM, 4 mM, 6 mM, 8 mM, 10 mM, and 110 mM ( ∼ 2%) glucose on survival of *S. cerevisiae* BY4741. As shown in Figure 2**A**, as the glucose concentration increases up to 10 mM, cell death increases. Interestingly, 10 mM and 110 mM glucose resulted in the same degree of SICD, as there was no significant difference in survival. These results support previous findings that show low concentrations of glucose can induce SICD 2, 4. More interestingly, a threshold effect was observed when the glucose concentration was above 10 mM.

Previous studies have shown that cells treated with 2% glucose (~110 mM) exhibit accumulation of intracellular reactive oxygen species (ROS) and internalize propidium iodide (PI) 3, 8, 10, 20. To determine if ROS is accumulating with treatment of 10 mM glucose, we stained cells with 2’,7’-dichlorodihydrofluorescein diacetate (H ${}_{2}$ DCFDA), which is a non-fluorescent compound that is converted to the fluorescent 2’,7’-dichlorofluorescein (DCF) upon oxidation by intracellular ROS. As shown in Figure 2**B**, cells in sorbitol are either unstained or lightly stained, however, 10 mM and 110 mM glucose resulted in cells fluorescing a brighter green than in sorbitol only, suggesting that ROS levels are increased. Treatment with glucose resulted in a significant increase in the population of cells staining for ROS (Figure 2**C**) and in the fluorescence intensity (Figure 2**D**) of stained cells, compared to treatment with sorbitol-only.

Similarly, cells were stained with Syto9 and PI to determine if there is membrane damage. Syto9 is a membrane-permeable dye that stains live cells green, while PI is a membrane-impermeable dye that enters the cell when the membrane is compromised, displacing Syto9 and causing the cells to stain red. As shown in Figure 2**B**, bottom, cells treated with 10 mM and 110 mM glucose stained with PI. There was a significant increase in PI-stained cells with 10 mM and 110 mM glucose treatments, compared to 0 mM glucose, but the increase is more dramatic with 110 mM glucose (Figure 2**E**). Together, these data suggest that 10 mM and 110 mM glucose may result in a similar cell death phenotype.

### *S. cerevisiae* BY4741 utilizes a variety of nitrogen sources

To determine the effect of nitrogen sources on SICD, we selected organic and inorganic nitrogen sources and first confirmed whether they can support the growth of *S. cerevisiae* BY4741, then determined their effect on SICD. The organic nitrogen sources were glutamate, glutamine, arginine, glycine, proline, and urea, while the inorganic nitrogen sources were (NH ${}_{4}$ ) ${}_{2}$ SO ${}_{2}$ and NH ${}_{4}$ Cl. It should be noted that glutamate, glutamine, arginine, (NH ${}_{4}$ ) ${}_{2}$ SO ${}_{\mathrm{4,}}$ and NH ${}_{4}$ Cl are typically referred to as preferred nitrogen sources, while glycine, proline, and urea are non-preferred nitrogen sources.

**Figure 3:**
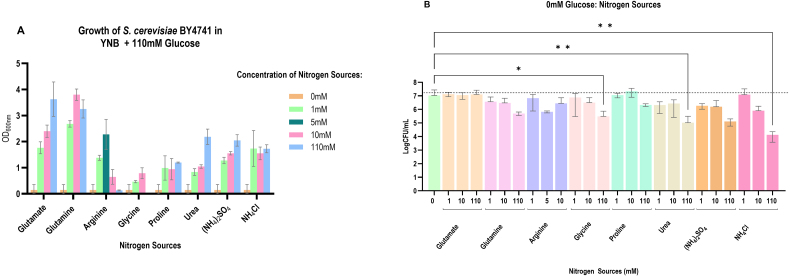
The effect of selected nitrogen sources on growth and survival. YNB medium with 110 mM (~2%) glucose was supplemented with 1 mM, 10 mM, and 110 mM of either glutamate, glutamine, glycine, proline, urea, (NH ${}_{4}$ ) ${}_{2}$ SO ${}_{4}$ , or NH ${}_{4}$ Cl, and was inoculated with *S. cerevisiae* BY4741 at the starting OD ${}_{\mathrm{600 nm}}$ of 0.01 **(A)**. The cultures were incubated at 30 ${}^{\circ }$ C and growth was determined after 48 hours of incubation. Arginine was also tested at 5 mM. The effect of the selected nitrogen sources on survival of *S. cerevisiae* BY4741 in sorbitol (0 mM Glucose) was determined by supplementing 2% sorbitol with 1 mM, 10 mM, and 110 mM of the selected nitrogen source and inoculating with an OD ${}_{\mathrm{600 nm}}$ of 1.3 cells **(B)**. The cultures were incubated at 37 ${}^{\circ }$ C for 72 hours and viability was determined. Arginine was tested at 1 mM, 5 mM, and 10 mM. The data shown are mean ± SD. The dashed line in B represents viability of the cells immediately after inoculation. ${}^{*}$ : *p* ≤ 0.014, ${}^{**}$ : *p* ≤ 0.003.

*S. cerevisiae* BY4741 was inoculated in YNB medium supplemented with the nutrients BY4741 is auxotrophic for and 110 mM glucose with either 0 mM, 1 mM, 10 mM, or 110 mM of the respective nitrogen sources. The cells were cultured for 48 hours at 30 ${}^{\circ }$ C. As shown in Figure 3, there was minimal growth in YNB only; however, supplementation with any of the nitrogen sources stimulated growth. Glutamate and glutamine were most effective at promoting growth, with as low as 1 mM resulting in a dramatic increase in the OD ${}_{\mathrm{600 nm}}$ . Arginine, on the other hand, was toxic at 10 mM and above, with maximum growth observed with 5 mM. For this reason, arginine will be studied under 1 mM, 5 mM, and 10 mM. Glycine, proline, urea, (NH ${}_{4}$ ) ${}_{2}$ SO ${}_{\mathrm{2,}}$ and NH ${}_{4}$ Cl stimulated growth, but were less effective than glutamate, glutamine, and arginine.

### A variety of nitrogen sources can support survival in the absence of glucose

Before we addressed the effect of nitrogen sources on SICD, we determined if the selected nitrogen sources exhibit any negative effect on survival in the absence of glucose. Arginine was assessed at 1 mM, 5 mM, and 10 mM. As shown in Figure 3**B**, survival of *S. cerevisiae* BY4741 in 1 mM and 10 mM of all the nitrogen sources was similar to that of sorbitol. Glutamate, glutamine, proline, and (NH ${}_{4}$ ) ${}_{2}$ SO ${}_{2}$ at 110 mM also resulted in similar survival to that in sorbitol. However, glycine, urea, and NH ${}_{4}$ Cl resulted in a 1-log, 2-log, and 3-log reduction in viability, respectively. Therefore, all the selected nitrogen sources can support survival; however, consistent with the literature 17, 18, at 110 mM concentration, a toxic effect is observed with inorganic and non-preferred nitrogen sources.

### Low concentrations of preferred organic nitrogen sources protect against SICD induced by 10 mM glucose

**Figure 4:**
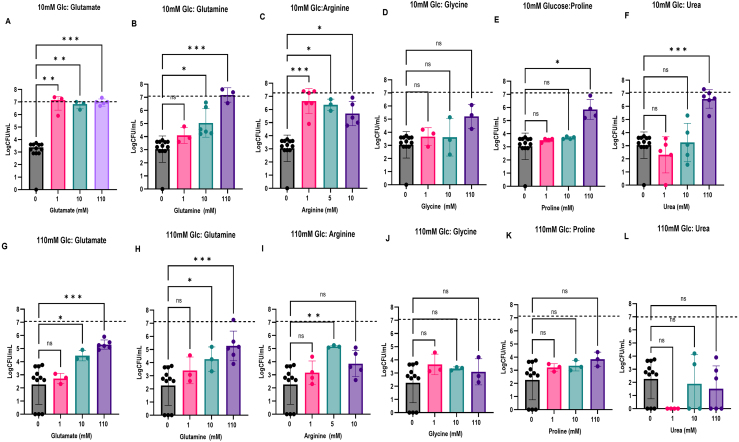
Organic nitrogen sources can protect against SICD induced by 10 mM glucose, but only highly preferred organic nitrogen sources can protect against SICD induced by 110 mM glucose. Glutamate, glutamine, glycine, proline, and urea at 1 mM, 10 mM, and 110 mM, and arginine at 1 mM, 5 mM, and 10 mM concentrations were supplemented to 2% sorbitol with either 10 mM glucose **(A–F)** or 110 mM glucose **(G–L)**. Survival was determined before incubation and after 72 hours of incubation at 37 ${}^{\circ }$ C. Survival was compared to that of cells in sorbitol only. The dashed lines represent viability of the cells immediately after inoculation. ${}^{*}$ : *p* ≤ 0.03, ${}^{**}$ : *p* ≤ 0.005,***: *p* ≤ 0.0006, ns: not significant.

Using 10 mM and 110 mM glucose to induce SICD, we tested the effect of 1 mM, 10 mM, and 110 mM of the selected organic nitrogen sources on SICD. Arginine was assessed at 1 mM, 5 mM and 10 mM. Cell viability was determined before incubation and 72 hours later. As shown in Figures 4**A**–**F**, in 10 mM glucose, there was a 4-log decline in viability; however, when 1 mM-110 mM glutamate was supplemented, viability was unchanged over the 72 hours of incubation (Figure 4**A**). Glutamine at 10 mM and 110 mM concentrations resulted in a 2-log and 4-log difference in viability, respectively, compared to 10 mM glucose only (Figure 4**B**). Arginine at 1 mM to 10 mM concentration resulted in almost a 3-log difference in viability, with 1 mM being the most protective (Figure 4**C**). Proline and urea protected against SICD at 110 mM concentration, with 3-log and 4-log difference in viability (Figures 4**E** and **F**), while glycine was not protective at any of the tested concentrations (Figure 4**D**). Therefore, low concentrations of glutamate, glutamine, and arginine, the highly preferred nitrogen sources, can protect against SICD induced by 10 mM glucose. It is noteworthy that 110 mM urea, which exerted a 2-log decline in viability without glucose (Figure 3**B**), did not result in a decrease in viability over the 72-hour period when supplemented with 10 mM glucose (Figure 4**F**).

When SICD was induced by 110 mM glucose, glutamate and glutamine, at 10 mM and 110 mM, and arginine at 5 mM, were protective against SICD, with a 2–3 log difference in viability (Figures 4**G**–**I**). On the other hand, glycine, proline, and urea failed to protect against SICD at all tested concentrations (Figures 4**J**–**L**).

Taken together, these findings suggest that low concentrations of preferred organic nitrogen sources and high concentrations of non-preferred organic nitrogen sources can prevent cell death induced by 10 mM glucose. However, cell death induced by 110 mM glucose can only be prevented by highly preferred organic nitrogen sources. Additionally, 110 mM glucose results in a more extreme phenotype, as it is more difficult to rescue by supplementation with nitrogen.

### Inorganic nitrogen sources cannot protect against SICD

We then determined whether inorganic nitrogen sources can also protect against SICD and if there is a different response depending upon the concentration of glucose present. We inoculated *S. cerevisiae* BY4741 in 10 mM and 110 mM glucose supplemented with 1 mM, 10 mM, and 110 mM (NH ${}_{4}$ ) ${}_{2}$ SO ${}_{4}$ or NH ${}_{4}$ Cl and determined cell viability before and after 72 hours of incubation.

**Figure 5:**
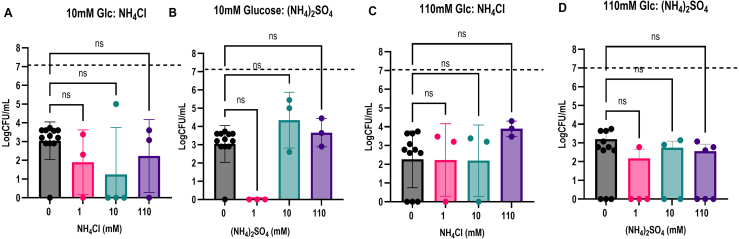
Inorganic nitrogen sources do not protect against SICD induced by 10 mM or 110 mM glucose. (NH ${}_{4}$ ) ${}_{2}$ SO ${}_{4}$ and NH ${}_{4}$ Cl at 1 mM, 10 mM, and 110 mM concentrations were supplemented to either 10 mM glucose (**A** and **B**) or 110 mM glucose (**C** and **D**), and viability was determined before incubation (0 hour) and 72 hours after incubation. The dashed lines represent viability before incubation. ns: not significant.

Interestingly, neither (NH ${}_{4}$ ) ${}_{2}$ SO ${}_{4}$ nor NH ${}_{4}$ Cl resulted in any significant difference in viability when compared to cells inoculated in 10 mM or 110 mM glucose only (Figure 5**A**–**D**). Even though both of these nitrogen sources are highly preferred, neither was protective against SICD, suggesting that organic nitrogen sources are more effective at preventing SICD.

**Figure 6:**
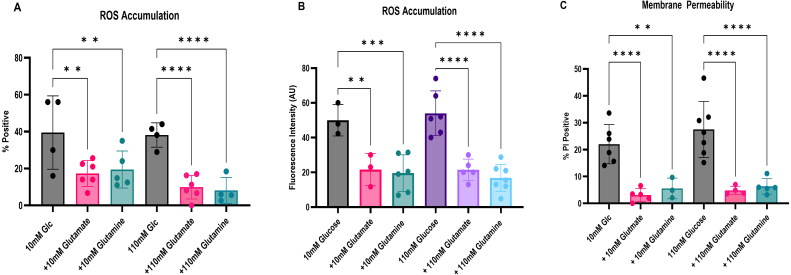
Glutamate and glutamine protect against SICD by reducing ROS levels and membrane damage. *S. cerevisiae* BY4741 was treated for an hour at 37 ${}^{\circ }$ C with 10 mM glucose and 10 mM of either glutamate or glutamine, and 110 mM glucose and either 110 mM glutamate or glutamine. The cells were stained with H ${}_{2}$ DCFDA to detect intracellular ROS, and PI and Syto9 to detect membrane damage. The percentage of cells staining positive for ROS **(A)** and fluorescence intensity **(B)** were determined for each treatment. The percentage of PI positive cells was also determined **(C)**. The data shown are mean ± SD. ${}^{**}$ : *p* ≤ 0.0068, ${}^{***}$ : *p* ≤ 0.0002, ${}^{****}$ : *p* < 0.00001.

### Glutamate and glutamine protected against SICD by reducing ROS accumulation and preventing membrane damage

Previous studies have shown that glutamate and glutamine protect against oxidative stress by enhancing resistance to ROS and extending the lifespan of *S. cerevisiae* 21–23*.* Glutamate and glutamine are also amine donors, which may facilitate amino acid biosynthesis 24, 25. To determine the role of glutamate and glutamine in protection against SICD, we treated cells with 10 mM or 110 mM glucose and a corresponding 10 mM or 110 mM of either glutamate or glutamine and checked for ROS accumulation and membrane damage. As shown in Figures 6**A** and **B**, glutamate and glutamine at 10 mM and 110 mM supplemented with 10 mM and 110 mM glucose, resulted in a significant reduction in the number of cells positive for ROS along with a significant reduction in fluorescence intensity of cells which stained with H ${}_{2}$ DCFDA. Similarly, there was a significant reduction in cells staining positive for PI, suggesting that there is a resulting decrease in membrane damage (Figure 6**C**). Therefore, glutamate and glutamine protected against SICD by reducing ROS accumulation and preserving membrane integrity.

### Extracellular glucose is not depleted during SICD

**Figure 7:**
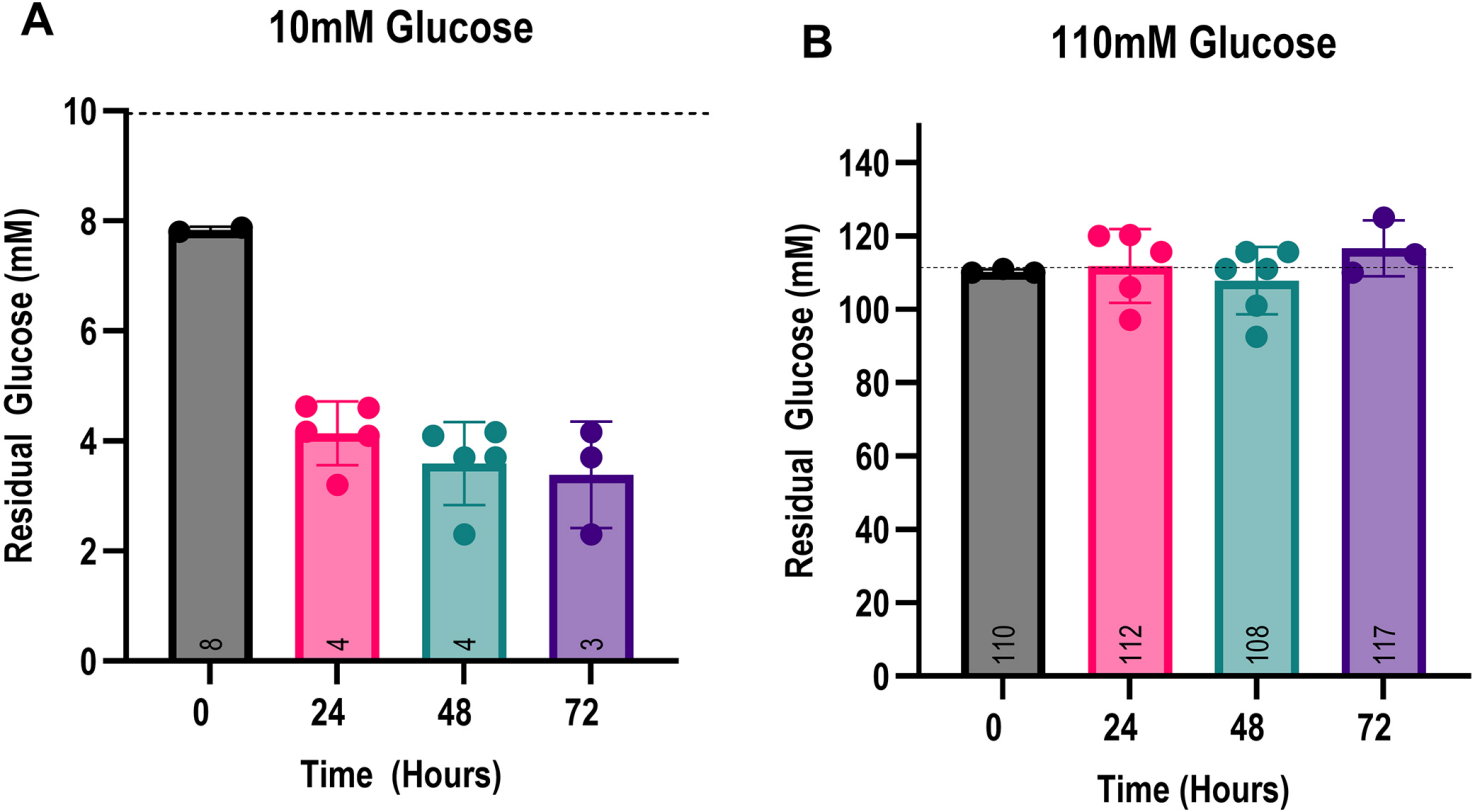
Glucose is not depleted during SICD. *S. cerevisiae* BY4741 was treated with 10 mM **(A)** or 110 mM **(B)** glucose at 37 ${}^{\circ }$ C. Cells were sampled at 0 hours and at 24-hour intervals for 72 hours. The glucose concentration in each sample was measured. The dashed line represents the initial concentration of glucose added to the cultures. The data shown are mean ± SD. The mean concentration of glucose at each time point is displayed in the respective bar.

One explanation for the decline in viability seen in *S. cerevisiae* during SICD is perhaps glucose is being depleted. To address this possibility, we measured the extracellular glucose concentration to determine if there is glucose exhaustion over time. The glucose concentration was measured using glucose oxidase to catalyze the oxidation of glucose to gluconic acid and H ${}_{2}$ O ${}_{2}$ . H ${}_{2}$ O ${}_{2}$ reacts with *o*-dianisidine in the presence of peroxidase to form a colored product and the absorption can be read at 540 nm using a spectrophotometer. As shown in Figure 7**A**, cells in 10 mM glucose cultures utilized about 2 mM of glucose immediately after inoculation and another 4 mM over the next 24 hours, then there was no further glucose utilization. However, in 110 mM glucose culture, the glucose concentration remained unchanged over the 72 hours, with some trials showing an increase in glucose concentration (Figure 7**B**). Taken together, these data suggest that glucose exhaustion is not the reason for cell death in SICD.

### Genetic modification of cell death machinery can protect against SICD

It was previously shown that when stationary phase cells are transferred to glucose-only solutions, the cell death phenotype resembles that of apoptosis 3, however, exponential phase cells undergo necrosis 14. Apoptosis in yeast can be caspase-dependent or independent 26. Previous studies have also shown that SICD in exponential phase and stationary phase cells is not dependent on the yeast metacaspase, *YCA1* 7, 8. Given the differential response observed in protection against SICD induced by 10 mM and 110 mM glucose, we investigated how cell death pathways are regulated under these conditions.

**Figure 8:**
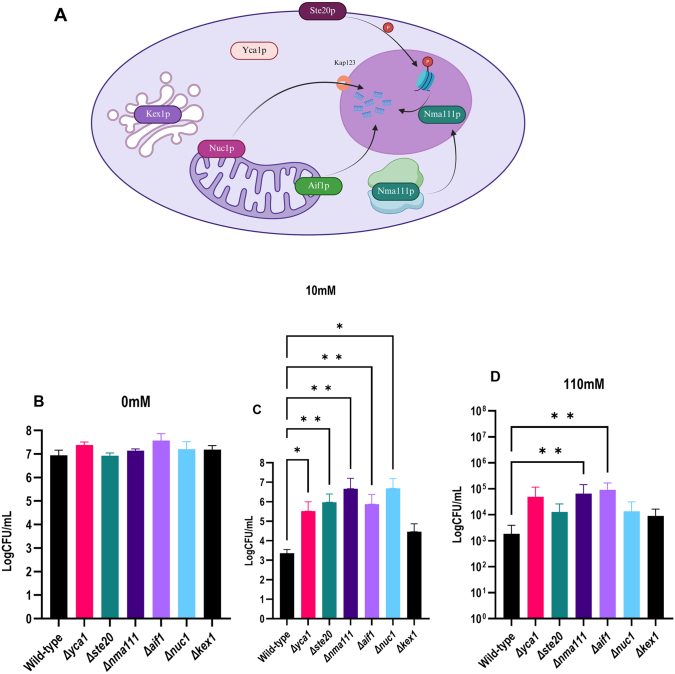
Genetic modification of cell death machinery protects against SICD. The cellular localization of proteins involved in caspase-dependent and -independent apoptosis is shown in **(A)**. Mutants deficient in *YCA1*, *AIF1*, *KEX1*, *NMA111*, *NUC1*, and *STE20* were collected and treated with 0 mM **(B)**, 10 mM **(C)** and 110 mM **(D)** glucose. Viability was determined before incubation and after 72 hours of incubation. The data shown is survival after 72 hours of incubation. ${}^{*}$ : p < 0.01, ${}^{**}$ : p < 0.006, ns: not signification. Figure 8**A** Created in BioRender. Parbhudayal, R. (2026) https://BioRender.com/xk5rgc7.

Commercially available deletion mutants of genes involved in caspase-independent apoptosis and the yeast metacaspase, *YCA1*, from the BY4741 parental strain were obtained and treated with 10 mM and 110 mM glucose. We monitored survival to determine if any of these deletions could protect against SICD. The mutants obtained were: Δ *yca1,* Δ *kex1,* Δ *nuc1,* Δ *aif1,* Δ *nma111* and Δ *ste20.* In the absence of caspase, the proteolytic activity of Kex1p, encoded by *KEX1*, was found to be important for cell death in response to defective N-glycosylation 27. *NUC1*, the yeast homolog of mammalian endonuclease G, and apoptosis-inducing factor (*AIF1*) proteins were shown to translocate from the mitochondria to the nucleus during cell death, leading to DNA fragmentation 28, 29. During cell death, protein levels of the nuclear mediator of apoptosis (Nma111p), encoded by *NMA111,* also increases in the nucleus 30 and phosphorylation of the histone 2B complex by Ste20p (encoded by *STE20*) occurs 12. Δ *ste20* was shown to confer resistance to cell death induced by H ${}_{2}$ O ${}_{2}$ 12 and glucose 11. The cellular localization of selected proteins is depicted in Figure 8**A**.

These mutants were obtained and treated with either 0 mM, 10 mM, or 110 mM glucose, and viability was determined at 0 hours and 72 hours. When the WT and mutants were incubated in 0 mM glucose, viability remained unchanged at 72 hours (Figure 8**B**); however, in the presence of 10 mM glucose (Figure 8**C**), survival of the WT decreased. Statistical analysis revealed that deletion of *YCA1*, *STE20*, *NMA111*, *AIF1*, and *NUC1* significantly protected cells from SICD, with an almost 3-log difference in survival compared to WT. Deletion of *KEX1,* on the other hand, did not protect against SICD.

Interestingly, in 110 mM glucose (Figure 8**D**), deletion of *NMA111* or *AIF1* was protective, resulting in a 2-log difference in viability compared to the WT. All other mutants were not protected. Therefore, it can be concluded that under low concentrations of glucose, SICD can be both caspase-dependent and -independent. While under higher concentrations of glucose, SICD appears to be caspase-independent. These findings are also consistent with our conclusion of SICD induced by 110 mM glucose being more extreme.

**Figure 9:**
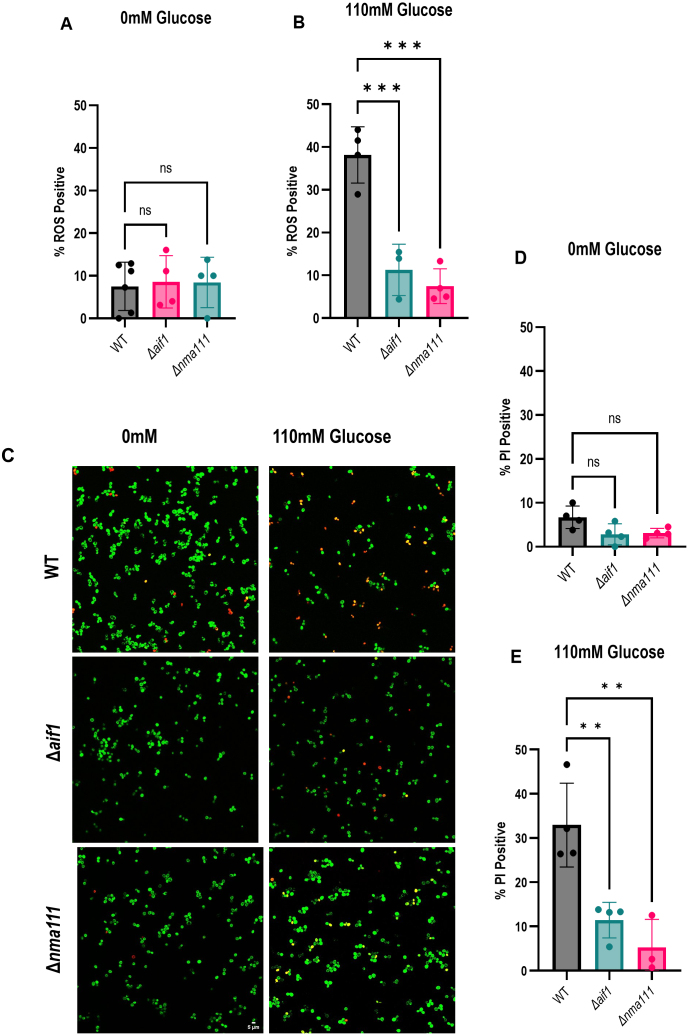
Δ aif1 and Δ nma111 shows reduced ROS level and membrane damage in SICD conditions. WT, Δ *aif1*, and Δ *nma111* were treated with sorbitol (0 mM glucose) or 110 mM glucose and incubated at 37 ${}^{\circ }$ C for one hour. Cells were then stained with H ${}_{2}$ DCFDA to detect intracellular ROS. The percentage of cells staining positive for ROS was determined for all strains in 2% sorbitol **(A)** and 110 mM glucose **(B)**. The cells were also stained with Syto9 and PI to detect membrane damage **(C)**. The percentage of PI positive cells were determined in sorbitol **(D)** and 110 mM glucose **(E)**. ${}^{**}$ : *p* ≤ 0.0004, ${}^{***}$ : *p* ≤ 0.0048, ns: not significant.

### Δ *aif1* and Δ *nma111* exhibit reduce ROS accumulation and membrane damage in glucose

Since Δ *aif1* and Δ *nma111* were protected against SICD, we investigated the levels of ROS in response to glucose. Δ *aif1,* Δ *nma111,* and the WT were treated with 110 mM glucose for 1 hour and stained for ROS. As shown in Figure 9, in sorbitol only, the ROS population remains similar in all strains (Figure 9**A**). However, in 110 mM glucose, there was 31% and 21% difference in ROS-positive population in Δ *aif1* and Δ *nma111*, respectively, compared to the WT (Figure 9**B**). We then stained the cells with Syto9 and PI to determine if these mutations also resulted in a decrease in membrane damage. As shown in Figure 9**C**, WT cells showed an increase in PI internalization in 110 mM glucose compared to sorbitol only, with an obvious decrease in the cell density in the field of view. Δ *aif1* and Δ *nma111*, on the other hand, showed decreased PI internalization compared to the WT. There was a 21% and 27% difference in PI population of Δ *aif1* and Δ *nma111*, respectively, compared to the WT (Figures 9**D** and **E**). Taken together, these findings suggest that Δ *aif1* and Δ *nma111* are protected due to the reduction of ROS levels and preservation of membrane integrity.

## DISCUSSION

One of the current theories as to why yeast cells die when exposed to glucose-only is that glucose acts as a signal for growth, however, in the absence of other nutrients to support growth, cell death is initiated 3, 4, 7, 9, 15. It was previously shown that nitrogen in the form of ammonium citrate and amino acids can suppress SICD, however, the names of the amino acids were not listed 1, 15. In this study, we investigated the impact of different nitrogen sources on SICD by first supplementing 2% (110 mM) glucose with components of minimal medium (YNB) and rich medium (YPD). We observed that the higher the nitrogen content was in YNB medium, the less protective it was against SICD (Figures 1**D** and **E**). On the other hand, complex nitrogen sources such as tryptone and yeast extract, protected against SICD (Figures 1**B** and 1**C**), perhaps because of the assortment of peptides and other compounds present, which created a balance to support survival.

It was previously shown that SICD can be induced by lower concentrations of glucose 2, 3. We speculated that high glucose concentration is masking the protective effect of nitrogen against SICD. To our surprise, we found that as glucose concentration increased up to 10 mM, cell death increased, however, 10 mM and 110 mM glucose resulted in the same extent of cell death (Figure 2**A**). Cells treated with 10 mM and 110 mM glucose also showed similar levels of ROS accumulation and membrane damage (Figures 2**B**–**E**).

We found that preferred organic nitrogen sources (glutamate, glutamine, and arginine), even at low concentrations, were protective against SICD induced by 10 mM glucose (Figures 4**A**–**C**), while non-preferred organic nitrogen sources (proline and urea) were protective at high concentrations only (Figures 4**E** and **F**). SICD induced with 110 mM glucose can only be prevented by preferred organic nitrogen sources (Figures 4**G**–**L**). Interestingly, a difference in the degree of protection against 10 mM and 110 mM glucose was observed. SICD induced by 10 mM glucose can be rescued completely by 1 mM glutamate or 1 mM arginine. However, a complete rescue was not observed by any of the nitrogen sources when SICD was induced by 110 mM glucose. Additionally, preferred inorganic nitrogen sources (NH ${}_{4}$ Cl and (NH ${}_{4}$ ) ${}_{2}$ SO ${}_{4}$ ) were not protective against SICD at any of the concentrations tested (Figures 5**A**–**D**). These findings suggest that organic nitrogen sources may be highly favored by cells when exposed to glucose and no other nutrients. This may be because during SICD, cells are not exposed to any other nutrients, and there is APT exhaustion 10, therefore, it is possible that utilization of NH ${}_{4}^{+}$ is not cost effective for the cell under such stressed conditions 24.

It is also likely that at 110 mM glucose, even though the extent of cell death is similar to that with 10 mM glucose, the mechanism may be more catastrophic. Similar observations were previously described in H ${}_{2}$ O ${}_{2}$ - and acetic acid-induced cell death in yeast, whereby low concentrations of the stimuli resulted in apoptosis while higher concentrations resulted in necrosis. 12, 31–33.

Glutamate and glutamine are fundamental players in several metabolic pathways in yeast. They are both precursors to glutathione synthesis, are involved in amino acid synthesis, and can be a nitrogen source for synthesis of nitrogenous compounds with intermediates of the tricarboxylic acid (TCA) cycle 21, 23, 34. Glutamate was shown to extend lifespan by enhancing resistance to intracellular ROS 21, 22. Deficiency in glutamate biosynthesis was shown to cause hypersensitivity to oxidative stress, leading to apoptosis 21. In our study, glutamate and glutamine were very effective at rescuing cells from SICD, with 1 mM of glutamate being sufficient to protect against 10 mM glucose (Figure 4**A**) and 10 mM glutamate or glutamine sufficient to rescue cells from 110 mM glucose (Figures 4**A, B, G,** and **H**). One possibility is that glutamate and glutamine serve as nitrogen donors to TCA intermediates to generate essential amino acids and other nitrogenous compounds. Additionally, glutamate may also participate in glutathione synthesis, relieving the cells from oxidative stress, thereby promoting cell viability. Glutamate and glutamine protected against SICD by lowering ROS accumulation and membrane damage (Figures 6**A, B,** and **C**), and there was no observed increase in CFU after 72 hours of incubation (Figures 4**A, B, G** and **H**), suggesting that glutamate and glutamine may be functioning to reduce oxidative stress as opposed to being involved in amino acid biosynthesis. However, further studies are needed for confirmation.

Arginine at 1 mM and 5 mM was very effective at rescuing cells from 10 mM and 110 mM glucose, respectively (Figures 4**C** and **I**). This is not surprising as arginine was previously reported to protect yeast, including *S. cerevisiae,* against ethanol-induced stress by reducing ROS levels, preserving cellular and mitochondrial membrane integrity, and activating nitric oxide synthesis, which results in activation of antioxidant defense systems 35, 36. Proline and urea, at 110 mM protected against SICD induced by 10 mM glucose (Figures 4**E** and **F**). While intracellular accumulation of proline in stationary phase cells of *S. cerevisiae* was shown to extend lifespan in YPD medium and induce antioxidant response in plants 34, 37, 38, there is limited work on the effect of urea on cell death in yeast. One study with synthetic defined (SD) medium with 2% glucose showed up to 37.5 mM of urea resulted in no change in cell viability for 18 days 16, while another study showed that urea protects renal medullary cells against NaCl-induced apoptosis 39.

Like glucose, ammonium was shown to induce cell death in *S. cerevisiae*. The cells undergo apoptosis followed by necrosis, especially when starved for auxotrophic nutrients 17, 18. In 2% to 10% glucose, ammonium reduced the chronological life span of *S. cerevisiae* in SD medium; however, in 0.5% glucose, this was not the case 16–18, 40, 41. In our current study, ammonium toxicity was also observed (Figure 3**B**). Additionally, ammonium did not protect against SICD, even though it is regarded as a preferred nitrogen source (Figures 5**A**–**D**).

It was previously shown that when stationary phase cells are transferred to glucose-only solutions, the cell death phenotype resembles that of apoptosis 3, however, exponential phase cells undergo necrosis 14. Apoptosis in yeast can be caspase-dependent or independent 7, 8, 26. We found that under 10 mM glucose, SICD can be rescued by deletion of genes involved in caspase-dependent and -independent cell death, except for *KEX1*. Interestingly, with 110 mM glucose, deletion of *AIF1* and *NMA111* rescued cell death while *YCA1*, *STE20*, *NUC1*, and *KEX1* deletion did not. Therefore, under 10 mM glucose, SICD can be caspase-dependent or caspase-independent, while under 110 mM glucose, cell death is preferentially caspase-independent. This represents the first study showing *YCA1* implication in SICD, however, it was only detected under 10 mM glucose and not the conventionally used 110 mM 7, 8.

Deletion of *AIF1* or *NMA111* protected against SICD induced by 110 mM glucose by decreasing ROS accumulation and preserving membrane integrity (Figures 9**A**–**D**). Aif1p is a mitochondrial-localized protein that is translocated to the nucleus upon induction of apoptosis. Δ *aif1* was also shown to be protected from H ${}_{2}$ O ${}_{2}$ and acetic acid-induced cell death, while the overexpression of *AIF1* increased cell death 28. *AIF1* and *YCA1* may have redundant functions in some forms of cell death, however, *AIF1* is involved in caspase-independent apoptosis 42, 43. The role of *AIF1* is also conserved in *Candida albicans,* as deletion of *AIF1* was shown to reduce apoptosis and necrosis, while overexpression was associated with increased apoptosis in *C. albicans* 44*.* Additionally, *AIF1* is also associated with response to antifungal drugs, as its deletion is implicated in resistance mechanisms 44–46. Consistent with our findings, protection against cell death by *AIF1* deletion in *C. albicans* was also associated with decreased ROS accumulation 44.

It was previously shown that Δ *nma111* is thermotolerant and does not exhibit apoptosis hallmarks such as accumulation of ROS, DNA fragmentation, or chromatin condensation when treated with H ${}_{2}$ O ${}_{2}$ 30, 47. Likewise, overexpression of *NMA1111* resulted in cell death with hallmarks of apoptosis 30. Similarly, protection against SICD by *NMA111* deletion was also associated with decreased ROS levels and preservation of membrane permeability (Figures 9**A**–**E**). The finding of Δ *nma111* being protected from SICD, even in 110 mM glucose, suggests that temperature may play an important role in SICD, raising the question of whether thermotolerant strains are resistant to SICD. Nevertheless, these findings suggest that Aifp and Nma111p nuclear localization is important for SICD.

Lastly, an interesting observation from this study was our finding of minimal glucose usage over the 72-hour incubation period (Figures 7**A** and **B**). This data is strikingly similar to what is observed in mammalian cells in hyperglycemia, whereby high glucose results in oxidative stress and apoptosis in various cell types 48–51. Given the similarities between yeast cell death pathways and higher eukaryotic cells, SICD may be a useful tool to study glucose signaling in hyperglycemic conditions.

In conclusion, 110 mM glucose resulted in a catastrophic cell death phenotype that is caspase-independent, which can be prevented by supplementation with highly preferred organic nitrogen sources. Under lower concentrations of glucose (10 mM), the same extent of cell death was observed, but cell death can be rescued by modification of caspase-dependent or caspase-independent signaling or through supplementation of low concentrations of preferred organic nitrogen sources or high concentrations of non-preferred nitrogen sources. Therefore, SICD induced by lower concentrations of glucose is highly regulated and can be blocked at multiple levels. However, under high concentrations of glucose, the cells are committed to undergo cell death.

## MATERIALS AND METHODS

### Strains and media

The strains used in the study are shown in Table 1.

**Table 1:** Strains used in this study .

**Species**	**Strains**	**Genotype**	**Source**
*Saccharomyces* *cerevisiae*	BY4741	*MATa, his3* Δ *1*, *leu*2 Δ , *met15* Δ *0*, *ura3* Δ *0*	Horizon Discovery

	YHL007C	*BY4741* Δ *ste20*	

	YOR197W	*BY4741* Δ *yca1*	

	YNL123W	*BY4741* Δ *nma111*	

YNR074C	*BY4741* Δ *aif1*

YJL208C	*BY4741* Δ *nuc1*

YGL203C	*BY4741* Δ *kex1*

YPD broth was prepared with 1% Yeast Extract (Difco), 2% Tryptone (Sigma-Aldrich), and 2% glucose (Sigma-Aldrich). YPD agar plates were prepared by adding 1.5% (w/v) agar to YPD broth. Yeast Nitrogen Base Medium (YNB) only, YNB + (NH ${}_{4}$ ) ${}_{2}$ SO ${}_{4}$ , and YNB + (NH ${}_{4}$ ) ${}_{2}$ SO ${}_{4}$ with amino acids were purchased from Sigma-Aldrich. A 10X stock solution was prepared and kept refrigerated. The amino acids present in YNB + (NH ${}_{4}$ ) ${}_{2}$ SO ${}_{4}$ with amino acids were histidine, tryptophan, and methionine, and were added by the manufacturer. 1M stock solutions of (NH ${}_{4}$ ) ${}_{2}$ SO ${}_{4}$ (Sigma-Aldrich), NH ${}_{4}$ Cl (Sigma-Aldrich), urea (Sigma-Aldrich), monosodium glutamate (MSG) (Sigma-Aldrich), and glycine (Sigma-Aldrich) were prepared in reverse osmosis (RO) water. Stock solutions of proline (Sigma-Aldrich), arginine (Sigma-Aldrich), and glutamine (Sigma-Aldrich) were prepared at 400 mM, 760 mM, and 200 mM, respectively, in distilled water. All solutions were filter-sterilized with a 0.22 μ m pore size filter purchased from ThermoFisher Scientific.

### SICD assays

For induction of SICD, *S. cerevisiae* BY4741 was cultured to stationary phase (incubated for 48 hours) in YPD medium at 30 ${}^{\circ }$ C with shaking at 200RPM. The cells were washed twice in autoclaved water and were diluted to OD ${}_{\mathrm{600 nm}}$ of 1.3 in either 2% sorbitol or 2% sorbitol + glucose at the desired concentrations and incubated at 37 ${}^{\circ }$ C for 72 hours with shaking at 200RPM. Sorbitol was used as an osmo-protectant. The average OD ${}_{\mathrm{600 nm}}$ of stationary phase cultures of the WT (BY4741), Δ *aif1,* and Δ *nma11* were 7.8, 9.8, and 8.0, respectively.

To test the effect of complex nitrogen sources on SICD, cells were inoculated in 2% glucose with 0%, 0.1%, 0.5%, 1% and 2% (w/v) yeast extract or tryptone. To test the effect of minimal medium on SICD, cells were incubated in either 2% glucose supplemented with either 1x or 1/10 ${}^{\mathrm{th}}$ YNB only, YNB + (NH ${}_{4}$ ) ${}_{2}$ SO ${}_{4}$ , or YNB + (NH ${}_{4}$ ) ${}_{2}$ SO ${}_{4}$ with amino acids, as indicated above.

To test the effect of organic and inorganic nitrogen sources on SICD, stationary phase cells were washed twice in autoclaved water and were inoculated in 2% sorbitol with 0 mM, 10 mM (0.18%) or 110 mM ( ∼ 2%) glucose, with 1 mM, 10 mM, or 110 mM of the respective nitrogen sources. The cultures were incubated at 37 ${}^{\circ }$ C with shaking at 200 RPM for 72 hours.

The number of viable cells was determined by serial dilutions in YPD medium using a 96-well plate, and the cells were plated on YPD agar plates. Sampling was performed at pre-incubation (0 hours) and after 72 hours of incubation at 37 ${}^{\circ }$ C. The plates were incubated at 30 ${}^{\circ }$ C for 48 hours, and then the number of colony-forming units (CFUs) was counted.

### Growth assays

To determine the effect of glutamate, glutamine, arginine, glycine, proline, urea, (NH ${}_{4}$ ) ${}_{2}$ SO ${}_{4}$ , and NH ${}_{4}$ Cl, on the growth of *S. cerevisiae* BY4741, 5 mL of YNB (supplemented with auxotrophic nutrients: uracil, methionine, histidine and leucine), with a total glucose concentration of 110 mM ( ∼ 2%) was inoculated with OD ${}_{\mathrm{600 nm}}$ 0.01 exponential phase cells, and they were incubated at 30 ${}^{\circ }$ C with shaking at 200RPM. The OD ${}_{\mathrm{600 nm}}$ was measured at 48 hours using a BIO-RAD SmartSpec ${}^{\mathrm{TM}}$ 3000 UV-Vis Spectrophotometer.

### Quantification of residual glucose

The residual glucose in the SICD culture supernatants was quantified using a Glucose Oxidase (GO) Activity Kit from Sigma-Aldrich (Product #: MAK501). Quantification was done using the manufacturer’s protocol. A standard curve was generated using the provided glucose standard, which generated an R-squared value of 0.9986. Supernatants were collected immediately upon inoculation of stationary phase *S. cerevisiae* BY4741 in 10 mM or 110 mM glucose (T = 0), and after 24, 48, and 72 hours after incubation at 37 ${}^{\circ }$ C. A 1:100 and 1:1000 dilution of the 10 mM and 110 mM culture supernatants were prepared, respectively, then 125 μ L was transferred to a 24-well plate with 250 μ L glucose oxidase reagent. The mixture was incubated at 37 ${}^{\circ }$ C for 30 minutes, then reaction was stopped with sulfuric acid. The absorbance of the samples was read at 540 nm using a BioTek Synergy H1 Plate reader.

### Detection of intracellular ROS

ROS was detected using 2’,7’-dichlorodihydrofluorescein diacetate (H ${}_{2}$ DCFDA) (Invitrogen ${}^{\mathrm{TM}}$ by ThermoFisher, Catalog # D399). A 10 mM working solution was prepared in ethanol daily for staining. The cells were treated for 1 hour then collected by centrifugation, washed in phosphate-buffered saline, pH 7.4, and stained with 10 μ M H ${}_{2}$ DCFDA for 30 minutes at 30 ${}^{\circ }$ C. The cells were visualized using a Leica SP5 Laser Confocal Microscope with a 63x water immersion objective lens. Images were obtained at 1x and 3x digital zoom and were analyzed with ImageJ 1.54p.

### Live/dead staining

Live/dead staining was performed using the Invitrogen ${}^{\mathrm{TM}}$ LIVE/DEAD *Bac*Light ${}^{\mathrm{TM}}$ Bacterial Viability Kit (Catalog # L7012), which contains the membrane-permeable STYO9 dye and the membrane-impermeable Propidium Iodide (PI). The dyes were resuspended in water at a 2X concentration, according to the manufacturer’s protocol. The cells were treated for 1 hour, centrifuged, resuspended in 50 μ L PBS, pH 7.4, and mixed with 50 μ L of the 2X LIVE/DEAD stain. The mixture was incubated at 30 ${}^{\circ }$ C for at least 15 minutes. Imaging was done using a Leica SP5 Laser Confocal Microscope with a 63x water immersion objective lens with sequential scanning at 488/50–530 nm (excitation/emission) for SYTO9 and 543 nm/604–700 nm for PI. Analysis was done with ImageJ 1.54p.

### Statistical analysis

Statistical analysis was done with GraphPad Prism 10. The data presented are mean with standard deviation (SD). A two-tailed unpaired t-test was done to compare growth of *S. cerevisiae* BY4741 at 30 ${}^{\circ }$ C and 37 ${}^{\circ }$ C. Survival was compared among groups using a Kruskal–Wallis test followed by Dunn’s multiple comparison test. *p* < 0.05 was considered statistically significant. Statistical analysis for quantification of ROS and PI-stained populations was done using Dunnett’s test to compare the control and treatments.

## CONFLICT OF INTEREST

The authors declare that there is no conflict of interest concerning the publication of this manuscript.

## ABBREVIATIONS

PI – propidium iodide

ROS – reactive oxygen species

SICD – sugar-induced cell death

TCA – tricarboxylic acid
